# Gender and Inconsistent Evaluations: A Mixed-methods Analysis of Feedback for Emergency Medicine Residents

**DOI:** 10.5811/westjem.58153

**Published:** 2023-08-22

**Authors:** Alexandra Brewer, Laura Nelson, Anna S. Mueller, Rebecca Ewert, Daniel M. O’Connor, Arjun Dayal, Vineet M. Arora

**Affiliations:** * University of Southern California: Los Angeles, Department of Sociology, Los Angeles, California; † University of British Columbia, Department of Sociology, Vancouver, British Columbia, Canada; ‡ Indiana University, Department of Sociology, Bloomington, Indiana; § Northwestern University, Chicago, Illinois; ∥ Massachusetts General Brigham Wentworth-Douglass Hospital, Dermatology and Skin Health, Dover, New Hampshire; ¶ Rush Copley Medical Group, Aurora, Illinois; # University of Chicago, Pritzker School of Medicine, Chicago, Illinois

## Abstract

**Objectives:**

Prior research has demonstrated that men and women emergency medicine (EM) residents receive similar numerical evaluations at the beginning of residency, but that women receive significantly lower scores than men in their final year. To better understand the emergence of this gender gap in evaluations we examined discrepancies between numerical scores and the sentiment of attached textual comments.

**Methods:**

This multicenter, longitudinal, retrospective cohort study took place at four geographically diverse academic EM training programs across the United States from July 1, 2013–July 1, 2015 using a real-time, mobile-based, direct-observation evaluation tool. We used complementary quantitative and qualitative methods to analyze 11,845 combined numerical and textual evaluations made by 151 attending physicians (94 men and 57 women) during real-time, direct observations of 202 residents (135 men and 67 women).

**Results:**

Numerical scores were more strongly positively correlated with positive sentiment of the textual comment for men (r = 0.38, *P* < 0.001) compared to women (r = −0.26, *P* < 0.04); more strongly negatively correlated with mixed (r = −0.39, *P* < 0.001) and negative (r = −0.46, *P* < 0.001) sentiment for men compared to women (r = −0.13, *P* < 0.28) for mixed sentiment (r = −0.22, *P* < 0.08) for negative; and women were around 11% more likely to receive positive comments alongside lower scores, and negative or mixed comments alongside higher scores. Additionally, on average, men received slightly more positive comments in postgraduate year (PGY)-3 than in PGY-1 and fewer mixed and negative comments, while women received fewer positive and negative comments in PGY-3 than PGY-1 and almost the same number of mixed comments.

**Conclusion:**

Women EM residents received more inconsistent evaluations than men EM residents at two levels: 1) inconsistency between numerical scores and sentiment of textual comments; and 2) inconsistency in the expected career trajectory of improvement over time. These findings reveal gender inequality in how attendings evaluate residents and suggest that attendings should be trained to provide all residents with feedback that is clear, consistent, and helpful, regardless of resident gender.

Population Health Research Capsule What do we already know about this issue?

*A gender gap in ACGME milestone attainment emerges over the course of Emergency Medicine (EM) residency, such that women receive significantly lower scores than men in their final year.*

What was the research question?

*Does resident gender influence how attending physicians score residents on ACGME milestone-based evaluations?*

What was the major finding of the study?

*Male residents received more evaluations where the numerical score and sentiment of the textual comment matched than female residents.*

How does this improve population health?

*This study identifies biases and gender inequalities in EM workforce training. A representative and well-trained EM workforce likely improves patient care and outcomes.*

## INTRODUCTION

Although women now graduate from medical school at the same rate as men,^1^ they remain underrepresented in academic medicine.^2^ The greatest attrition of women from academic medicine occurs directly after residency,^1^ pointing to the importance of gender dynamics taking place within graduate medical education to the production of long-term inequality in men’s and women’s medical careers. Several recent studies have examined how gender bias in residency may have consequences for women’s pursuit of academic careers and persistence in academic medicine.^3^^–^^6^ Medicine is a male-typed field^3^^,^^7^: even as women are increasingly represented among physicians, there are still strong cultural associations between men and the role of doctor, which can lead to implicit bias against women physicians.^8^ Associations between men and doctoring may be particularly strong in emergency medicine (EM), which is among the most male-dominated medical specialties.^9^^,^^10^

A recent study from our group found that a gender gap in Accreditation Council for Graduate Medical Education (ACGME) milestone attainment appears to emerge over the course of EM residency, with attendings scoring male residents higher than female residents.^5^ This attainment gap was not dependent on the gender of the attending physician doing the evaluation, or the gender pairing between the attending and resident.^5^ Research building on this study has used the textual comments attached to numerical scores to unpack how gender bias may have influenced attendings’ numerical scores. Qualitative studies analyzing the textual comments attached to attendings’ numerical scores of resident performance have found that EM faculty give women residents less consistent feedback,^6^ feedback that is more focused on personality than clinical competence,^6^ harsher criticism,^3^ and less helpful and reassuring feedback^3^ than to males.

While our prior work focused on either numerical or textual evaluations, with this study our goal was to examine concordance between numerical scores and textual comments to better understand gender gaps in resident evaluations in the emergency department. Does gender bias influence how attendings physicians assign residents numerical scores? Greater discordance between textual comments and numerical scores for women than men suggest gender bias may shape the way feedback is provided, which in turn may contribute to the established gender gap in evaluations and may harm resident education.

## METHODS

We conducted a mixed methods analysis of textual and numerical ACGME milestone-based evaluations of EM residents by attending physicians.

### Data Collection

We collected study data from four geographically diverse, three-year ACGME-accredited, EM training programs at university hospitals between July 1, 2013–July 1, 2015. Training programs were included in this study if they had adopted InstantEval V2.0 (Monte Carlo Software LLC, Annandale VA), a direct-observation mobile app for collecting ACGME milestone evaluations, and if they enabled faculty to leave both numerical and textual evaluations.

We analyzed a total of 11,845 evaluations by 151 attendings (94 men and 57 women) of 202 residents (135 men and 67 women) across all three years of residency. Each evaluation consisted of a numerical ACGME Emergency Medicine Milestone Project-based performance level (1, 1.5, 2, 2.5, 3, 3.5, 4, 4.5, or 5) on 1 of 23 possible individual EM subcompetencies,^11^ along with an optional text comment (limited to 1,000 characters). The application presented faculty members with descriptors of individual milestones and the meaning of numerical performance levels as they performed the evaluation. Faculty were supposed to assess one milestone per evaluation. A score of 1 was to be assigned in cases where the resident performed at the skill level expected of an incoming resident; 2 when the resident was improving but not yet performing at mid-residency level; 3 when the resident had mastered most of the skills expected of a graduating resident; 4 when the resident was performing at the skill level expected of a graduating resident; and 5 when the resident was performing beyond the level expected of a graduating resident.^11^ Most programs encouraged 1–3 evaluations per shift, but faculty could choose when to complete evaluations, whom to evaluate, and how many evaluations to complete.

### Data Analysis

Our analytic strategy combined complementary quantitative and qualitative methods.^12^ Quantitative methods allowed us to identify broad patterns in comment type and scores across our data, while we used qualitative methods to confirm that the mechanisms suggested by the quantitative methods (ie, inconsistent evaluations) were explicitly apparent in the comments received by the actual residents in our data.

First, all textual comments from all four hospitals were qualitatively coded in NVivo 12 (QST International, Burlington, MA), a qualitative analysis software package. Four team members conducted focused coding of comments for positive (containing only praise), negative (containing only criticism), mixed (both positive and negative), or neutral (containing neither praise nor criticism) tone and content. See examples in Table 1. To ensure consistency, at least two of four team members coded every comment, and any discrepancies between codes were discussed by all four team members until a consensus was reached. Information about residents’ and faculty members’ gender was hidden during qualitative coding to prevent confirmation bias. This process was imperfect since some comments included gendered pronouns or names of residents. While we tried using sentiment analysis techniques to identify valence, we found that the standard techniques did not capture the true valence of the words in our data. For example, *patient* is considered a positive word (as in the adjective related to patience) in most sentiment dictionaries, but it is a neutral word in our data (noun *patients*). We thus found hand-coding the most reliable and accurate method to identify comment valence.

**Table 1.** Definitions of Positive, Negative, Mixed, and Neutral Codes.

Code	Definition	Example
Positive	Comment contains praise of resident and no criticism	Resident is truly above his level of training. He is complete and thoughtful in his treatment of patients and understands the overall goal. Performed very well with a difficult intubation.
Negative	Comment contains criticism of resident and no praise	I hope that this is the first rotation in the ED, otherwise he is cleary runing behind his peers. Needs a lot of prompting, lacks of initiative and insight, looks scared, poor follow up on his own patients. Performance below average.
Mixed	Comment contains both praise and criticism	Good data gathering, but needs to work on differtial diagnosis. Also, instaead of asking questions needs to take some responsability to figure things out, look things up.
Neutral	Comment contains neither praise and criticism	Worked to place ultrasound guided lines this month.

**Table 2.** Descriptive statistics by postgraduate year and resident gender in four hospitals.

	PGY1				PGY2				PGY3			
	Women		Men		Women		Men		Women		Men	
	34		71		43		79		33		70	
Number of residents evaluated	Mean	Std. Dev.	Mean	Std. Dev.	Mean	Std. Dev.	Mean	Std. Dev.	Mean	Std. Dev.	Mean	Std. Dev.
Number of Comments per Resident	37	22	36	17	35	21	39	21	32	22	34	19
Positive Comments per Resident	29	18	25	14	27	16	31	18	23	18	27	17
Mixed Commments per Resident	5	5	7	5	5	5	5	5	4	5	3	4
Negative Comments per Resident	3	5	3	4	3	4	2	3	1	3	1	2
Score by Resident	2.3	0.2	2.3	0.2	2.7	0.2	2.7	0.2	2.9	0.3	3.0	0.3

Note: Our unit is resident/year. These numbers include duplicate counts of residents who were evaluated over multiple years. There were 64 residents who were evaluated in both their first and second years of the program, and 64 who were evaluated in both their second and third years, for a total of 330 resident/year units and 202 unique residents.

*PGY*, postgraduate year.

In our second, quantitative step, we calculated the total number of comments for each valence type and the overall average score across all comments for each resident in all four hospitals. To measure the relationship between comment valence and numerical score, we correlated the total number of positive, mixed, and negative comments with average score by resident gender, reporting the Pearson correlation coefficient and its associated *P*-value as an indication of the magnitude and significance of the correlation. To measure comment type over the course of residency training, we counted the number of positive, mixed, and negative comments by resident gender and by their year in the program and tabulated the average counts by resident gender in all four hospitals.

Third, we conducted a final round of detailed qualitative coding to assess whether patterns in the quantitative results were likely to be meaningful to residents. During this stage, we focused on a single hospital site chosen because it was the largest (in terms of number of attendings, residents, and evaluations) of the four sites in our dataset. All examples of comments in the text come from this single site. Three team members coded all 3,120 text comments from this site as either consistent or inconsistent with numerical score. Consistency was defined as a fit in sentiment between textual comments and numerical scores, eg, a positive comment was attached to a higher-than-average numerical score, and inconsistency as a misfit. Three analysts conducted a single round of coding on PGY-3 data from the other three hospitals to ensure this one hospital was not idiosyncratic and found it was not.

All names used in the text are pseudonyms to protect confidentiality. Since numerical scores have different meanings for residents at different stages of training, we present qualitative examples of PGY-1 residents in the results for the sake of simplicity. This study was approved as exempt research by the University of Chicago Institutional Review Board.

## RESULTS

We found that attendings’ assessments of female residents were less consistent than those of male residents. Inconsistency occurred at two levels: 1) inconsistency between numerical scores and textual comments; and 2) inconsistency in the expected career trajectory.

### 1. Inconsistency between numerical score and textual comment

**Figure 1.**
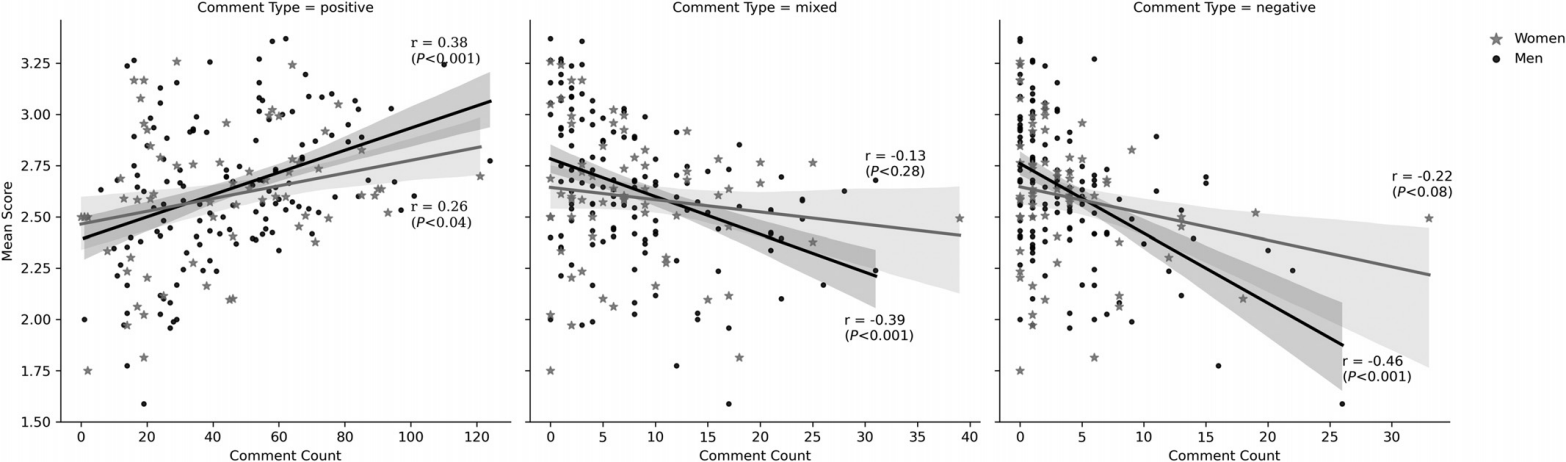
Scatter plot with regression line, correlation coefficient (and *P*-value) for number of comments and mean score, by resident gender and comment type at four hospitals. *Note:* r is Pearson correlation coefficient (and *P*-value), by resident gender and comment type. The shaded ribbons represent the 95% confidence interval.

We found that for both men and women residents, there was a positive correlation between average evaluation score and the number of positive comments the resident received, and a negative correlation between the average score and number of mixed and negative comments. However, this relationship was stronger for comments of all valences for men, with a Pearson correlation coefficient of 0.26 (and a *P*-value < 0.04) for positive comments and score for women compared to 0.38 for men (<0.001), −0.22 (<0.08) for negative comments and score for women and −0.46 (<0.001) for men, and −0.13 (<0.28) for mixed comments and score for women compared to −0.39 (<0.001) for men. For women, the correlation between mixed and negative comments and score was not statistically significant (see Figure 1).

In-depth qualitative analysis from one hospital revealed that textual comments that matched numerical scores provided clarity as to what residents were doing well and where they needed to improve. Consider the following positive comment written by attending Steven for resident Eugene: “Really continues to come along nicely. Good follow up and follow through for results, status, and disposition.” This comment was associated with a numerical score of 3.5 out of 5, indicating that Steven thought that intern Eugene was performing at mid-residency level.

Conversely, men received lower average scores when they received more negative comments compared to women across all four hospitals in our sample (Figure 1), again indicative of greater consistency in their feedback from attendings. In our qualitative analysis of a single site, the following comments made by attendings Michael and Greg for residents Zander and Spencer, both of which were accompanied by a numerical score of 1 of 5 (the lowest possible numerical score, indicating that residents were not yet performing at the level expected of an incoming intern), were typical of consistent negative feedback.

• “As we discussed during and after shift, reviewing the electronic medical record can provide significant useful information to guide your management. Take a few moments to look for discharge summaries or recent ED visits.” (Michael for Zander)
• “Continue to work on building a list of differentials for each patient. This will help you not only figure out what studies you want to order but also what questions you need to ask to help narrow it down.” (Greg for Spencer)

On the other hand, attendings were more inconsistent in how they assessed women’s performances, assigning them a higher number of numerical scores that mismatched their textual comments. Our qualitative analysis showed that when it came to positive comments with low scores, attendings often simultaneously communicated that women were succeeding and failing at the same skill. For instance, attending Harrison wrote about intern Josie, “Managed a patient well with cuts and abrasions, sutures small lac, seemed to have reasonable skills for her level.” While this comment contains modest praise for Josie’s ability to manage wounds, Harrison assigned Josie a score of 1 of 5 for this comment, a rating meant to indicate a lower-than-intern skill level.

This pattern was similar for positive scores assigned to negative comments. In one typical example, attending Megan gave resident Kendall a score of 3 of 5, indicating that she was performing at mid-residency level in intern year, but criticized her ability to formulate a plan for patient care, stating, “Seems to hesitate with forming final plan/dispo for patient, presents patient knowing the problem but doesn’t always have a clear plan of care in mind.” Similarly, attending Allison told resident Jade, “Needs to start taking [the] next step forward. ‘I’m just an intern’ will not play for much longer.” Despite this criticism, Allison assigned her a 3.5 of 5, indicating that her abilities were well above intern level.

Our data suggests that ≈11% more women than men (26 of 67 women, and 42 of 135 men) received overall “inconsistent” feedback between textual comments and score, measured by residents receiving a higher-than-average number of comments by comment valence and either a lower-than-average score (for positive comments) or a higher-than-average score (for mixed and negative comments).

### 2. Inconsistency in expected career trajectory

The ACGME EM guidelines outline a clear trajectory whereby residents are expected to gradually improve over the course of residency through the attainment of skills in 23 subcompetencies. Their recommendations for assigning numerical scores to resident progress reflect this idea of linear progress over time.

**Figure 2.**
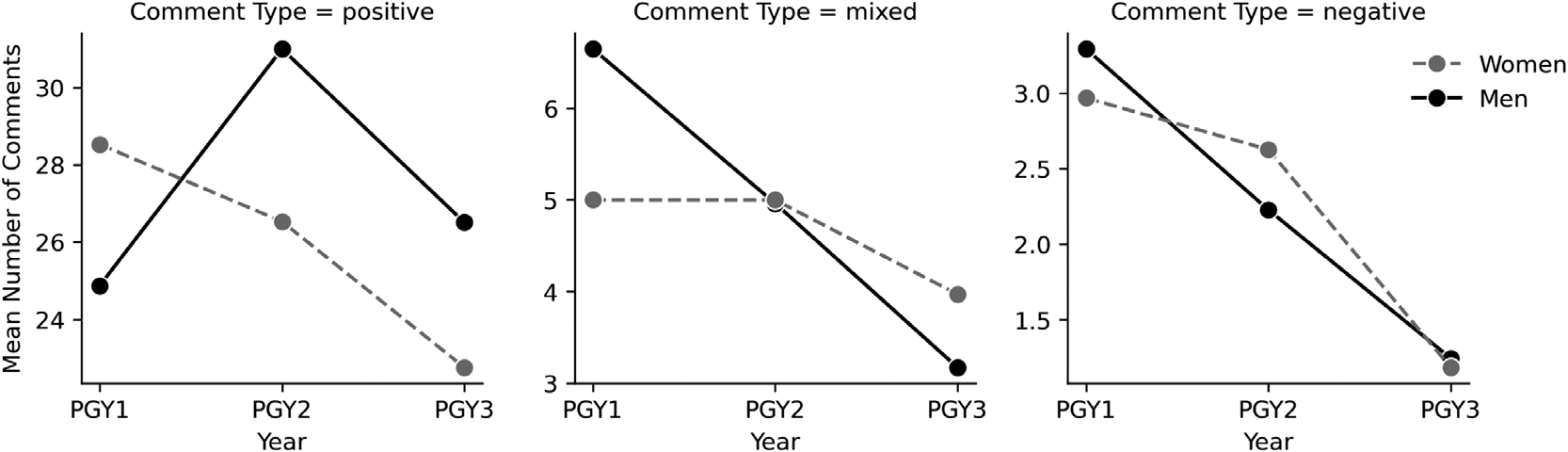
Mean number of comments by resident gender, postgraduate year, and comment type at four hospitals. *PGY*, postgraduate year.

Our analysis shows that men and women residents’ numerical scores are consistent with this expected pattern. Attendings gave both male and female residents higher numerical scores for their performance in PGY-3 (men received an average score of 3.0 and women 2.9) than in PGY-1 (both men and women received an average score of 2.3). Figure 2 visualizes the different patterns in textual sentiment for men and women residents from PGY-1 to PGY-3. Male residents received more negative and mixed comments in PGY-1 (an average of three negative comments and seven mixed comments) than in PGY-3 (one negative and three mixed), when they received a larger portion of positive comments. (Men received an average of 25 positive comments in PGY-1 and 27 in PGY-3.) Although male residents appear to be assessed more harshly than their female peers in PGY-1 in that they receive more negative and mixed comments, their trajectory over time is consistent with expectations of improvement as one advances through residency. Female residents, on the other hand, received more positive comments in PGY-1 and fewer in PGY-3 (an average of 29 positive comments in PGY-1 and 23 in PGY-3) while they received almost the same number of mixed comments on average in PGY-1 and PGY-3 (five compared to four). Both men and women received fewer negative comments in PGY-3 (one compared to three in PGY-1), although the number of negative comments overall is low.

## DISCUSSION

Our goal for this study was to examine the relationship between numerical and textual performance evaluations for EM residents to shed light on the findings of a prior quantitative study^5^ that demonstrated a gender gap in milestone attainment during EM residency. To do this, we used complementary quantitative and qualitative methods to analyze 11,845 numerical and textual evaluations made by 151 attending physicians during real-time direct observations of 202 residents. We identified two forms of inconsistency between numerical scores and textual performance evaluations that may have relevance to women’s lagging numerical scores on ACGME milestones and to gender inequality in residency.

First, the data revealed that women’s numerical scores were less consistent with the sentiment of their textual comments than those of men. Women residents in our sample were more likely to receive positive comments alongside lower scores, and negative or mixed comments alongside higher scores. Second, the data showed that textual comments for women, but not men, were inconsistent with the expected EM career trajectory of improvement over time. On average, men received slightly more positive comments in PGY-3 than in PGY-1 and fewer mixed and negative comments, while women received fewer positive and negative comments in PGY-3 than PGY-1 and almost the same number of mixed comments.

Inconsistent performance evaluations are worthy of attention for several reasons. First, inconsistent evaluations may harm women residents’ development as physicians. Critical feedback, when constructive, can be a source of valuable advice for improving one’s performance.^13^^,^^14^ Our qualitative findings demonstrate that consistency between numerical score and textual comment can offer useful information to residents by providing a clear explanation of why they earned a particular score and, on occasion, actionable feedback about how to improve in the future. Inconsistent feedback could be a barrier to learning from errors and to developing skills as a physician, especially in the earlier stages of residency, and could ultimately contribute to the gender gap in ACGME milestone attainment that emerges over the course of residency.^5^

Additionally, inconsistent evaluations may harm women’s confidence in their ability as physicians and/or their commitment to academic medicine. Several studies suggest that women in medicine suffer from imposter syndrome, the psychological phenomenon whereby people doubt their abilities even in the face of evidence of their own success.^15^^,^^16^ Inconsistent feedback could be a factor that contributes to gender disparities in feelings of imposter syndrome during residency: If women encounter more praise that is combined with criticism, this could foster doubt in their abilities as physicians. Further, prior research from the social sciences shows that confidence in one’s skills is critical for the persistence of women in male-typed professions like EM.^17^^,^^18^

Inconsistent feedback could be something that contributes to women’s attrition from academic EM, which occurs at a higher rate than that of men.^19^ The fact that women in our sample received fewer positive textual comments as they progressed from PGY-1 to PGY-3 may also be something that harms their confidence and that could have a broader impact on their careers. Computational simulations have suggested that even very small biases in everyday interactions, such as those we find here, can compound in complex systems to produce much larger patterns of inequality in organizations.^20^ Further study is warranted to examine the connection between inconsistent evaluations and attrition from medical careers, whether through imposter syndrome or another mechanism.

This study provides further support for the idea that gender bias contributes to the gender gap in evaluations by showing that attending physicians evaluate residents differently based on gender, with a bias in favor of men, even in the context of objective criteria such as the ACGME milestones. Based on these findings, we caution against the use of competency-based graduation from residency, even when programs rely on clearly articulated standards such as the ACGME milestones. Movement to competency-based graduation would likely disadvantage women residents and deepen inequalities in the medical profession.

## LIMITATIONS

There are limitations to our study. First, we did not have information about the broader context in which evaluations were written, including conversations that may have taken place between attendings and residents that could have provided additional information explaining numerical scores and/or textual feedback. Second, we did not have data about the race or ethnicity of the physicians in our sample, which may be a pertinent covariate given evidence of bias against people of color in the medical profession.^21^ Third, since our data was collected in 2013-15, it is possible that gender dynamics in residency programs may have changed in the intervening years, especially as residency programs have invested in reducing biases in training. Fourth, while we took several steps to guard against gender bias in our coding procedures – including using multiple coders, requiring coding consensus, and suppressing attending and resident gender – we did not suppress all gendered language in the textual comments. This may have biased our analysis. Fifth, since we only analyze data from four university hospitals, our findings may not be nationally representative of EM training programs in the US.

Our study also has several strengths. First, faculty did not know that their evaluations would be analyzed for gender bias, diminishing a Hawthorne effect or social desirability bias, and giving us a window into real conditions of gender inequality in medical education. Second, drawing on data from four university hospitals across the US allowed us to be more confident that the inequalities we found were not specific to one hospital environment, but rather may be generalizable across different organizational environments.

Future research is needed to examine the full scope of inconsistent feedback in graduate medical education, including commentary given to residents in person, as well as the consequences of inconsistent feedback for physicians’ careers, including attrition from medicine and feelings of burnout and imposter syndrome.

## CONCLUSION

By pairing quantitative and qualitative methods, this study contributes to research on gender inequality in graduate medical education by showing multiple levels at which women receive inconsistent feedback in residency. Initiatives to reduce gender inequality in medicine should train faculty to offer all residents clear, consistent, and helpful assessments of their performance, regardless of resident gender.
